# Genomic diversity, antibiotic resistance, and virulence in South African *Enterococcus faecalis* and *Enterococcus lactis* isolates

**DOI:** 10.1007/s11274-024-04098-5

**Published:** 2024-08-05

**Authors:** Oluwaseyi Samuel Olanrewaju, Lesego G. Molale-Tom, Cornelius C. Bezuidenhout

**Affiliations:** Unit for Environmental Sciences and Management, North-West University, Potchefstroom Campus, Private Bag X6001, Potchefstroom, 2520 South Africa

**Keywords:** Antibiotic resistance, One health, *Enterococcus faecalis*, Pangenome analysis

## Abstract

This study presents the empirical findings of an in-depth genomic analysis of *Enterococcus faecalis* and *Enterococcus lactis* isolates from South Africa. It offers valuable insights into their genetic characteristics and their significant implications for public health. The study uncovers nuanced variations in the gene content of these isolates, despite their similar GC contents, providing a comprehensive view of the evolutionary diversity within the species. Genomic islands are identified, particularly in *E. faecalis*, emphasizing its propensity for horizontal gene transfer and genetic diversity, especially in terms of antibiotic resistance genes. Pangenome analysis reveals the existence of a core genome, accounting for a modest proportion of the total genes, with 2157 core genes, 1164 shell genes, and 4638 cloud genes out of 7959 genes in 52 South African *E. faecalis* genomes (2 from this study, 49 south Africa genomes downloaded from NCBI, and *E. faecalis* reference genome). Detecting large-scale genomic rearrangements, including chromosomal inversions, underscores the dynamic nature of bacterial genomes and their role in generating genetic diversity. The study uncovers an array of antibiotic resistance genes, with trimethoprim, tetracycline, glycopeptide, and multidrug resistance genes prevalent, raising concerns about the effectiveness of antibiotic treatment. Virulence gene profiling unveils a diverse repertoire of factors contributing to pathogenicity, encompassing adhesion, biofilm formation, stress resistance, and tissue damage. These empirical findings provide indispensable insights into these bacteria’s genomic dynamics, antibiotic resistance mechanisms, and virulence potential, underlining the pressing need to address antibiotic resistance and implement robust control measures.

**Supplementary Information:**

The online version contains supplementary material available at 10.1007/s11274-024-04098-5.

## Introduction

Antibiotic-resistant bacteria have been detected in several environmental sources (Amarasiri et al. 2020; Bezuidenhout et al. 2023; Hassoun-Kheir et al. 2020; Kritzinger et al. 2023; Nnadozie and Odume 2019), and have become a significant global public health concern, increasing morbidity and mortality rates. The emergence and spread of antibiotic resistance seriously threaten medical treatment effectiveness for infectious diseases (Szadkowska et al. 2022). Antibiotic-resistant bacteria are responsible for hundreds of thousands of deaths yearly. The problem of antibiotic resistance is expected to worsen, with estimates suggesting that by 2050, 10 million people will die annually from antibiotic-resistant bacteria (Zhao et al. 2022). The development of antibiotic resistance in bacteria is a complex process that involves various mechanisms. Bacteria have evolved multiple strategies to become resistant to antibiotics, including alterations in bacterial proteins that are targeted by antibiotics, enzymatic degradation of antibiotics, changes in membrane permeability to reduce antibiotic entry, increased efflux of antibiotics, alterations of antibiotic-activating enzymes, and activation of resistant metabolic pathways (Parker et al. 2020). Additionally, the genetic basis of antibiotic resistance can be intrinsic or acquired, with acquired resistance often facilitated by the horizontal transfer of antibiotic-resistance genes among bacteria (Su et al. 2020; Zhang et al. 2023). To fight the spread of antibiotic resistance, wastewater treatment plants (WWTPs) are set up to control the spread of resistant bacteria. WWTPs help improve distributed water for various purposes. However, studies have reported concerns over the efficiency of the WWTPs in producing safe water for distribution effectively (Bezuidenhout et al. 2023; Kritzinger et al. 2023; Molale-Tom et al. 2024; Olanrewaju et al. 2024). Furthermore, because of the different sources of water in the WWTPs, there is a need for proper monitoring of these plants for safe water distribution.

Among the most widely studied antibiotic-resistant bacteria is the *Enterococcus faecalis*. These species and its closely related species, *Enterococcus lactis*, have been reported in various environments. *E. faecalis* and *E. lactis* possess virulence factors contributing to their pathogenicity. *E. faecalis* has been extensively studied for its pathogenic potential. It produces various virulence factors, including lipoteichoic acid, peptidoglycan, aggregation substance, cytolysin, and lytic enzymes (Dai et al. 2022). These factors enable *E. faecalis* to colonize and cause infections in various host environments. Biofilm formation is an important virulence trait of *E. faecalis*. It has been shown that *E. faecalis* can form biofilms, contributing to its persistence and resistance to antimicrobial agents (Zheng et al. 2020). Additionally, *E. faecalis* has been implicated in refractory apical periodontitis, a persistent infection of the root canal system, where it induces macrophage necroptosis, leading to the progression of the disease (Dai et al. 2022). The presence of virulence genes in *Enterococcus* species has also been investigated. A study on *Enterococcus* species isolated from yaks found that *E. faecalis* strains had a higher frequency of biofilm formation and virulence genes than other enterococcal species (Anna Woźniak-Biel 2019). The potential pathogenicity of *E. faecalis* has been further explored through genomic and functional characterization. A study analyzed the genomic characteristics of *Enterococcus* spp. isolated from a wastewater treatment plant and found that *E. faecalis* harbored virulence genes involved in adhesion, invasion, and sex pheromones (Mbanga et al. 2021). Genomic analysis of *E. faecalis* isolates recovered from the International Space Station revealed the presence of genes associated with pathogenicity, including those involved in adhesion, biofilm formation, and antibiotic resistance (Bryan et al. 2021).

We therefore hypothesize that whole-genome sequence, as one of the latest biotechnological tools, would be able to detect the antibiotic-resistant genes and virulence factors of the pathogenic *E. faecalis* and *E. lactis*. Therefore, this study aimed to identify the virulence factors and antibiotic-resistance gene profile through whole-genome sequencing of the water-pathogenic *E. faecalis* strains and commensal *E. lactis* strains isolated from WWTPs in South Africa. We further performed a pangenome analysis on the South African isolates of *E. faecalis* and comparative genomics to identify the prevailing antibiotic-resistant genes and virulence genes in South African isolates of *E. faecalis*.

## Materials and methods

### Isolation and genome sequencing

### Sample collection and bacterial isolation

Three WWTPs in the North West Province, which receive wastewater from urban households, industries, farms, and hospitals, were sampled. All samples were collected at the final effluent, points downstream, and a point between two of the WWTPs. The dip sampling technique was employed at each site as described by the United States Environmental Protection Agency (http://www.dem.ri.gov/pubs/sops/wmsr2013.pdf). Membrane filtration was employed for *Enterococcus* isolation and enumeration. KF-Streptococcus agar plates supplemented with Triphenyltetrazolium chloride (TTC) were incubated at 37 °C for 48 h. Single well-isolated pink colonies were aseptically sub-cultured three times on nutrient agar using the streak plate technique and incubated for 24 h at 37 °C.

The DNA was extracted using the Macherey-Nagel kit (Duren, Germany) following the manufacturer’s protocol. The NanoDrop-800 spectrophotometer (Thermo Fisher Scientific, Wilmington, NC, USA) and Qubit (ThermoFisher Scientific, US) quantified the gDNA following the manufacturer’s protocol. The paired-end Illumina library was prepared using Nextera XT Library Preparation kit (Illumina, US) and sequenced for (2 × 300 bp) cycles on Illumina MiSeq. Briefly, tagment genomic DNA was simultaneously fragmented and then tagged with adapter sequences in a single step using Nextera transposome (Nextera XT DNA Library Preparation Kit, Illumina, San Diego, CA, USA). Tagmented DNA was then amplified using a limited-cycle (12-cycle) PCR program. To purify the library DNA, amplified DNA was cleaned with AMPure XP beads. Then, Nextera library was quantified using Qubit, and the size profile was determined on the Agilent Technology 2100 Bioanalyzer using a high-sensitivity DNA chip (Agilent Technologies, Waldbronn, Germany). The library for sequencing was normalized to 1nM and pooled. Then, the 1nM pooled library was diluted and NaOH-denatured before loading for the sequencing run on a MiSeq sequencer (MiSeq reagent kit V2-300 cycles).

### Assembly and annotation

The raw paired-end fastq reads (2 × 300 bp) were quality-checked using FastQC v.0.11.7 (Andrews 2010) followed by trimming of low-quality bases using Trimmomatic v.0.39 (Bolger et al. 2014) and quality-checked again using FastQC v.0.11.7. The cleaned reads were assembled using SPAdes v.3.15.5 (Bankevich et al. 2012). To evaluate the quality of the genome assemblies, Quast (v.5.0.2) (Gurevich et al. 2013) was used, and completeness and contamination were assessed using CheckM (v.1.1.6) (Parks et al. 2015). Default settings were used in all tools except where otherwise stated. Further genomic analysis, annotation, and comparative genomics studies were carried out using these assemblies. The assembled draft genomes were annotated using the Rapid Annotation System Technology (RAST) Pipeline (Aziz et al. 2008). The genome and its typical features were visualized using proksee (v 1.1.2) (Grant et al. 2023), and the RAST subsystems for all isolates were viewed using circos (Krzywinski et al. 2009).

The GenBank accession numbers for L4_6M, L16_61, LTM1, and LTM3 are JAWJDK000000000, JAWKDV000000000, JAWJDI000000000, and JAWJDJ000000000 while the BioProject numbers are PRJNA1027583, PRJNA1027576, PRJNA1027578, and PRJNA1027580, respectively.

### Genome-based phylogenetic analysis

The genome sequence was uploaded to the Type (Strain) Genome Server (TYGS) at https://tygs.dsmz.de, for a whole genome and proteome-based taxonomic analysis (Meier-Kolthoff and Göker 2019). All user genomes were compared against all type strain genomes available in the TYGS database via the MASH algorithm (Ondov et al. 2016), and the ten type strains with the smallest MASH distances were chosen per user genome. An additional set of ten closely related type strains was determined via the 16 S rDNA gene sequences, which were extracted using RNAmmer (Lagesen et al. 2007). Each sequence was subsequently BLASTed (Camacho et al. 2009) against the 16 S rDNA gene sequence of each currently 19,225 type strain available in the TYGS database. Digital DDH values and confidence intervals were calculated using the recommended settings of the GGDC 3.0 (Meier-Kolthoff et al. 2013). The resulting intergenomic distances were used to infer a balanced minimum evolution tree with branch support via FASTME 2.1.6.1, including the SPR postprocessing (Lefort et al. 2015). Branch support was inferred from 100 pseudo-bootstrap replicates each, and the trees were rooted at the midpoint and visualized with PhyD3 (Kreft et al. 2017).

### Type-based species and subspecies clustering

The type-based species clustering using a 70% dDDH radius around each of the 29 type strains was done as previously described (Meier-Kolthoff and Göker 2019). Subspecies clustering was done using a 79% dDDH threshold as previously introduced (Meier-Kolthoff et al. 2014). In addition, the in silico DDH value was calculated by the Genome-to-Genome distance calculator (GGDC) to compare the genome. The phylogenetic tree was constructed based on the average nucleotide identity (ANI). The overall similarity between the whole-genome sequences was calculated using the Orthologous Average Nucleotide Identity Tool (OAT) v0.93.1 (Yoon et al. 2017).

### Analysis of genes associated with antimicrobial resistance, virulence, and secondary metabolites

The genomes were mined for biosynthetic gene clusters of antimicrobial compounds, including NRPs, PKs, NRPs-PKs hybrids, bacteriocins, and terpenes, with RAST system (Aziz et al. 2008). Antimicrobial resistance genes were mined using the Resistance Gene Identifier (RGI) tool of the Comprehensive Antibiotic Resistance Database (CARD) (Alcock et al. 2019) using contigs file with the parameters “Perfect and Strict hits only” and “High quality/coverage”. Default settings were used in all analyses except where otherwise stated. VirulenceFinder 2.0 was used to identify virulence-associated genes (Joensen et al. 2014). Identification thresholds were set 90% over a minimum identity length of 60%. Genome Islands were viewed using Islandviewer 4 (Bertelli et al. 2017).

### Pangenome and comparative genomics analysis

Pangenome analysis between South African *E. faecalis* isolates to reveal core, dispensable, and unique genes was conducted using Roary (Page et al. 2015) and Anvi’o (Eren et al. 2015). According to Roary, protein sequences with annotation were loaded, and an all-against-all BLASTP was used to cluster proteins. The sequence identity of over 95% was set as the threshold for clustering protein homologs. Anvi’o clustered homologs based on the similarity of amino acid sequences; the anvil-display-pan function generated the interactive visualization of results. Furthermore, we conducted a comparative analysis of the antibiotic-resistant and virulence genes to determine the variations in the occurrence of antibiotic-resistant and virulence genes in the *E. faecalis* isolates. We could not perform a pangenome analysis or a comprehensive antibiotic resistance and virulence gene analysis on the *E. lactis* genomes because of the relatively few available South African *E. lactis* genomes in the NCBI database.

## Results and discussion

Enterococci infection is a growing public health concern in South Africa and other African regions (Ali et al. 2018). Enterococci are becoming multidrug-resistant organisms, posing challenges for effective treatment. Studies have shown a prevalence of enterococci infections in different healthcare settings, including hospitals and primary-care facilities (Mogokotleng et al. 2023; Olawale et al. 2011). The prevalence of enterococci infections in South Africa has been analyzed through retrospective studies, identifying enterococci isolated from bloodstream infection samples (Mogokotleng et al. 2023). These studies provide valuable insights into the country’s prevalence and characteristics of enterococci infections. The emergence of vancomycin-resistant enterococci (VRE) is particularly concerning in South Africa (Foka et al. 2018). VRE has been found in various ecological niches, highlighting the need for effective control measures to prevent the spread of these resistant strains. The limited therapeutic options for VRE infections further emphasize the urgency of addressing this issue (Foka et al. 2018).

### Genome assembly and annotation

The size of the assembled *E. faecalis* and *E. lactis* genomes ranged from 2,657,678 bp to 2,996,468 bp (Table 1). The GC contents of these species’ genomes were not significantly different, ranging from 38.2 to 38.3% for the *E. faecalis* and 37.2–37.3% for the *E. lactis* species. The RAST annotation predicted coding sequences were 2679 and 2894 for *E. faecalis*, while for *E. lactis* were 2877 and 3002. The observed variation could be due to the evolution-based genetic diversity of the species. The constructed genomes generally exhibited satisfactory gene completeness levels (Table 1) and were a dependable resource for further analysis. Figure S1 shows the circular view of the four genomes in this study.

**Table 1:** Properties of the studied genomes

	L16_61	LTM1	L4_6M	LTM3
Size (bp)	2,840,958	2,657,678	2,978,862	2,996,468
GC content (%)	38.3	38.2	37.3	37.2
N50	200,147	113,357	436,385	434,843
L50	7	9	3	3
No. of contigs	94	89	299	71
No. of subsystems	226	219	249	245
No. of coding sequences	2894	2679	3002	2877
No. of RNAs	65	67	62	55
Completeness	99.63	99.63	99.63	99.63
Contamination	0.91	1.59	3.63	2.72

**Fig. 1:**
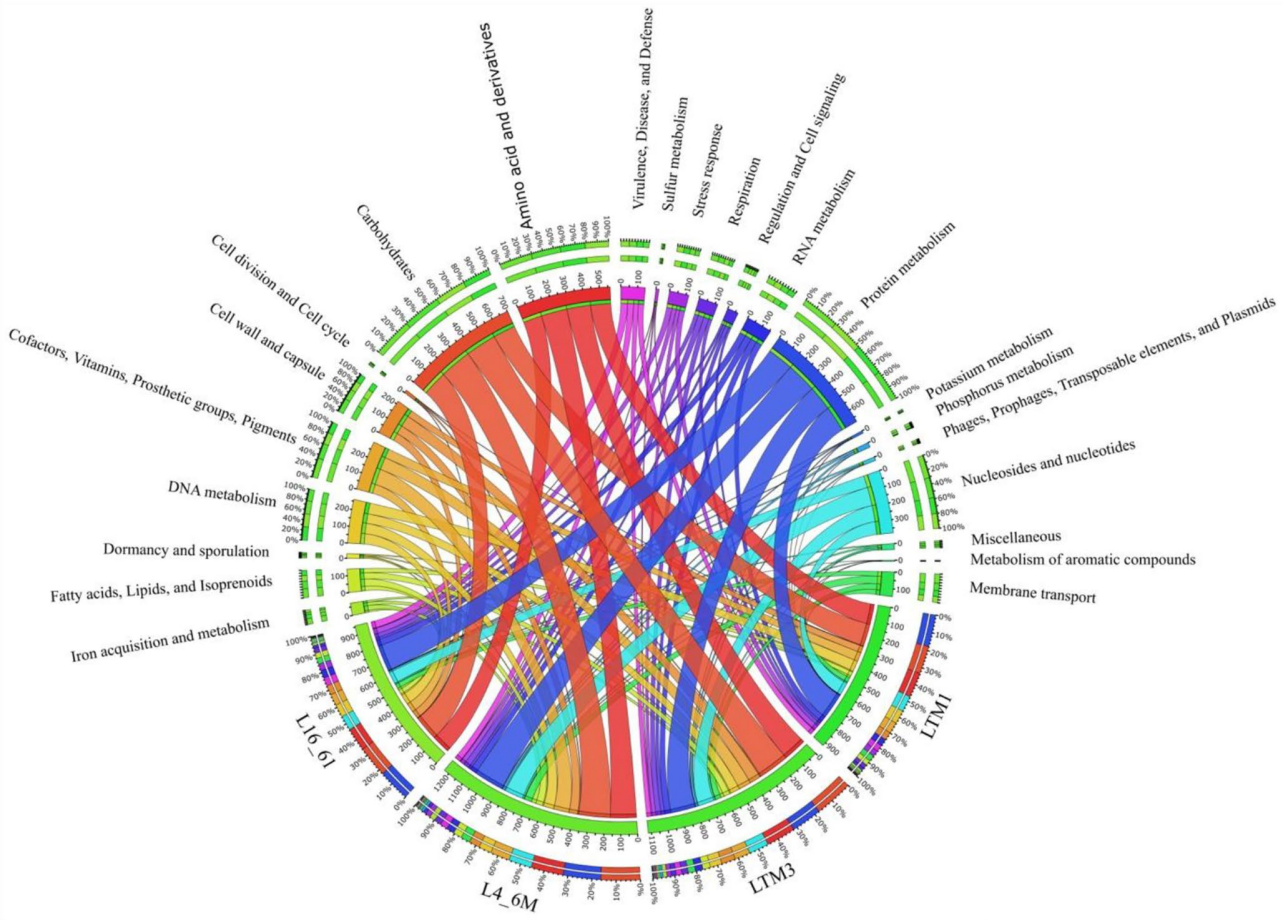
Functional overview of the isolate’s RAST subsystem annotations

The circos plot shows the RAST subsystem feature count distribution (Fig. 1). Noticeably, for L16_61, L4_6M, LTM1, and LTM3, RAST demonstrated the involvement of 43, 38, 34, and 30 genes in virulence, disease, and defense, respectively. Furthermore, RAST showed 35, 27, 32, and 23 genes in L16_61, L4_6M, LTM1, and LTM3, respectively, related to stress responses, including cold and heat shock, oxidative stress, osmotic stress, and detoxification, which help the strains to adapt to changing environmental conditions (Tables S1-S4). Spores and biofilm formation genes are also present in these genomes. *E. faecalis* and *E. lactis* are known to form biofilms, complex communities of microorganisms embedded in a self-produced extracellular matrix. Biofilm formation is a crucial virulence factor for these bacteria, allowing them to adhere to surfaces and resist host immune responses and antimicrobial treatments (Duggan and Sedgley 2007; Khalifa et al. 2016). Several studies have characterized virulence factors and genes associated with biofilm formation (Eaton and Gasson 2001; Zheng et al. 2017). In addition to biofilm formation, sporulation is another important aspect of the life cycle of enterococci. Sporulation is a survival mechanism that allows bacteria to form highly resistant spores under unfavorable conditions.

### Molecular identification and phylogenomics

To determine the taxonomic positions of the isolates, the digital DNA-DNA hybridization values were calculated between the isolates and selected close strains, which were determined by the TYGS algorithm. The dDDH values between the whole genome sequences are reported in Tables S5 and S6. These values of the strains in our study and the represented genomes are higher than 60% (DDH) for the conspecific assignation (Table S6).

**Fig. 2:**
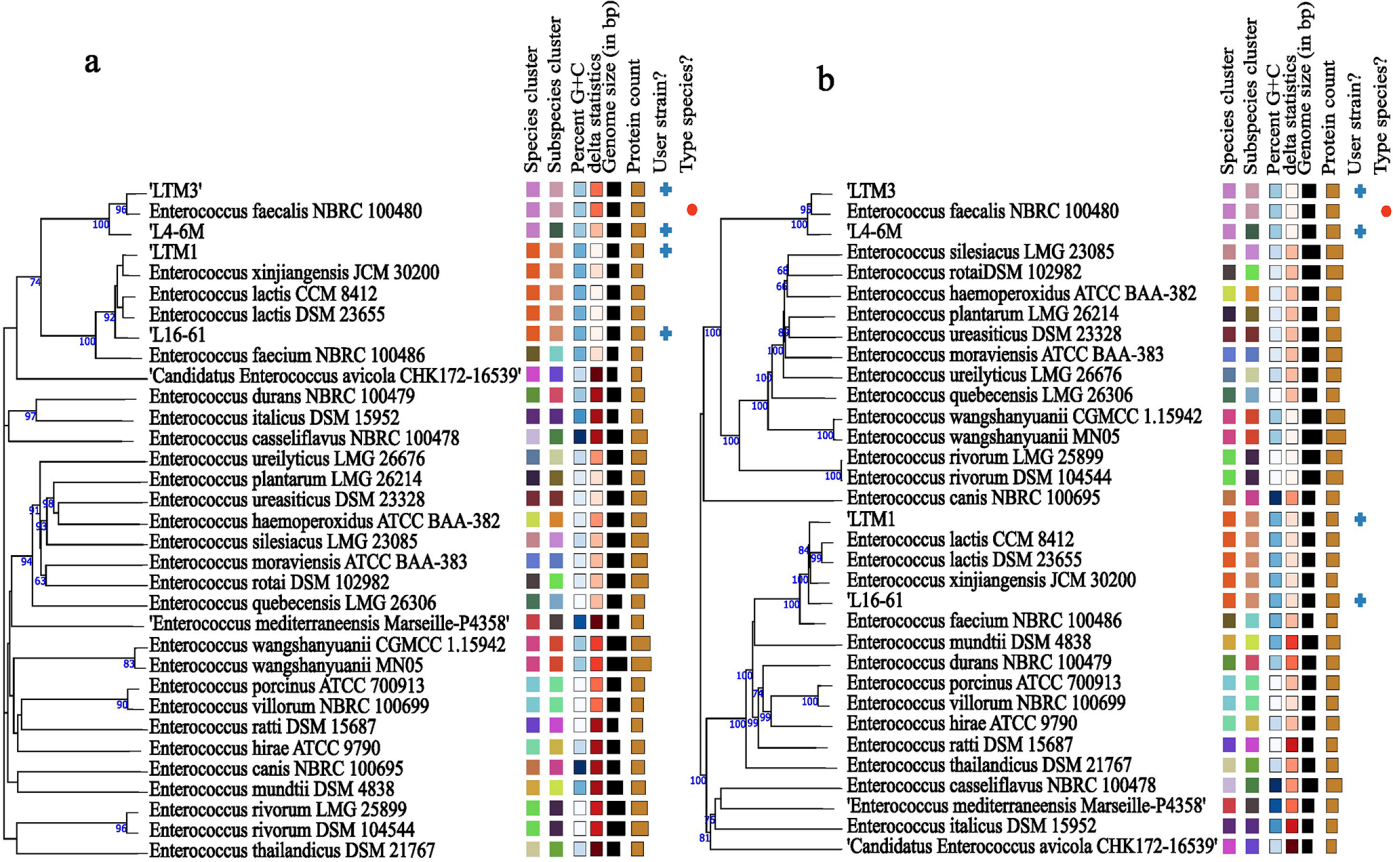
TYGS phylogenomics report. (**a**) genome-based phylogeny - Tree inferred from GBDP distances calculated from genome sequences. The branch lengths are scaled in terms of the GBDP distance formula d5. The numbers above branches are GBDP pseudo-bootstrap support values > 60% from 100 replications, with an average branch support of 58.6%. The tree was rooted at the midpoint. (**b**) proteome-based phylogeny- Tree inferred from whole-proteome-based GBDP distances. The branch lengths are scaled via the GBDP distance formula d5. Branch values are GBDP pseudo-bootstrap support values > 60% from 100 replications, with an average branch support of 84.5%. The tree was midpoint rooted

The phylogenomic tree based on the whole genome and proteome sequences is shown in Fig. 2, reconstructed on the TYGS server, showing the district phylogenetic positions of the *E. faecalis* and the *E. lactis* strains in the *Enterococcus* genus. The phylogenetic tree provided further evidence for the taxonomic position of the strains in the genus *Enterococcus*. Therefore, all the above analyses proved that the strains belonged to *E. faecalis* and *E. lactis*.

### Identification of Genomic Islands and Resistance genes

Genomic islands (GIs) are regions of bacterial genomes that have been acquired through horizontal gene transfer and often contain genes associated with specific functions, such as virulence, antibiotic resistance, or metabolic pathways (Hudson et al. 2014; Wei et al. 2016). These regions can play a significant role in bacterial evolution and adaptation to different environments. HGT is a well-established mechanism that can play a significant role in bacterial evolution and adaptation to different environments (Power et al. 2021; Virolle et al. 2020; Yu et al. 2023). HGT allows bacteria to acquire new genetic material from other bacteria or archaea, expanding their gene repertoire and potentially providing advantageous traits for survival and adaptation (Schönknecht et al. 2013; Touchon et al. 2017). This process has been observed to facilitate the origins of bacterial diversity, including diversity based on antibiotic resistance (Wiedenbeck and Cohan 2011). Genes acquired through HGT have been implicated in rapidly adapting bacteria to novel environments (Lawrence and Ochman 1998). Environmental adaptation is facilitated by horizontal gene transfer from various bacteria and archaea, followed by gene family expansion (Schönknecht et al. 2013). Conjugation, a form of HGT, has been found to play a profound role in bacterial evolution by spreading genes that allow bacteria to adapt to and colonize new niches (Leonetti et al. 2015). HGT has also been shown to speed up the adaptation of bacteria to new ecological niches and mitigate the genetic load of clonal reproduction (Power et al. 2021). Phage-mediated HGT has been identified as a mechanism that diversifies microbial gene repertoires and contributes to bacterial adaptation (Touchon et al. 2017). Recombination, which includes HGT, is recognized as a central source of variation for adaptive evolution in many species of bacteria (Levin and Cornejo 2009). Conjugation drives the rapid evolution and adaptation of bacterial strains by mediating the propagation of various metabolic properties, including symbiotic lifestyle, virulence, biofilm formation, and resistance to antibiotics (Virolle et al. 2020). Bacterial adaptation to extreme environments is often mediated by HGT (Yu et al. 2023). HGT is a dynamic process that can significantly impact bacterial evolution and adaptation to different environments.

**Fig. 3:**
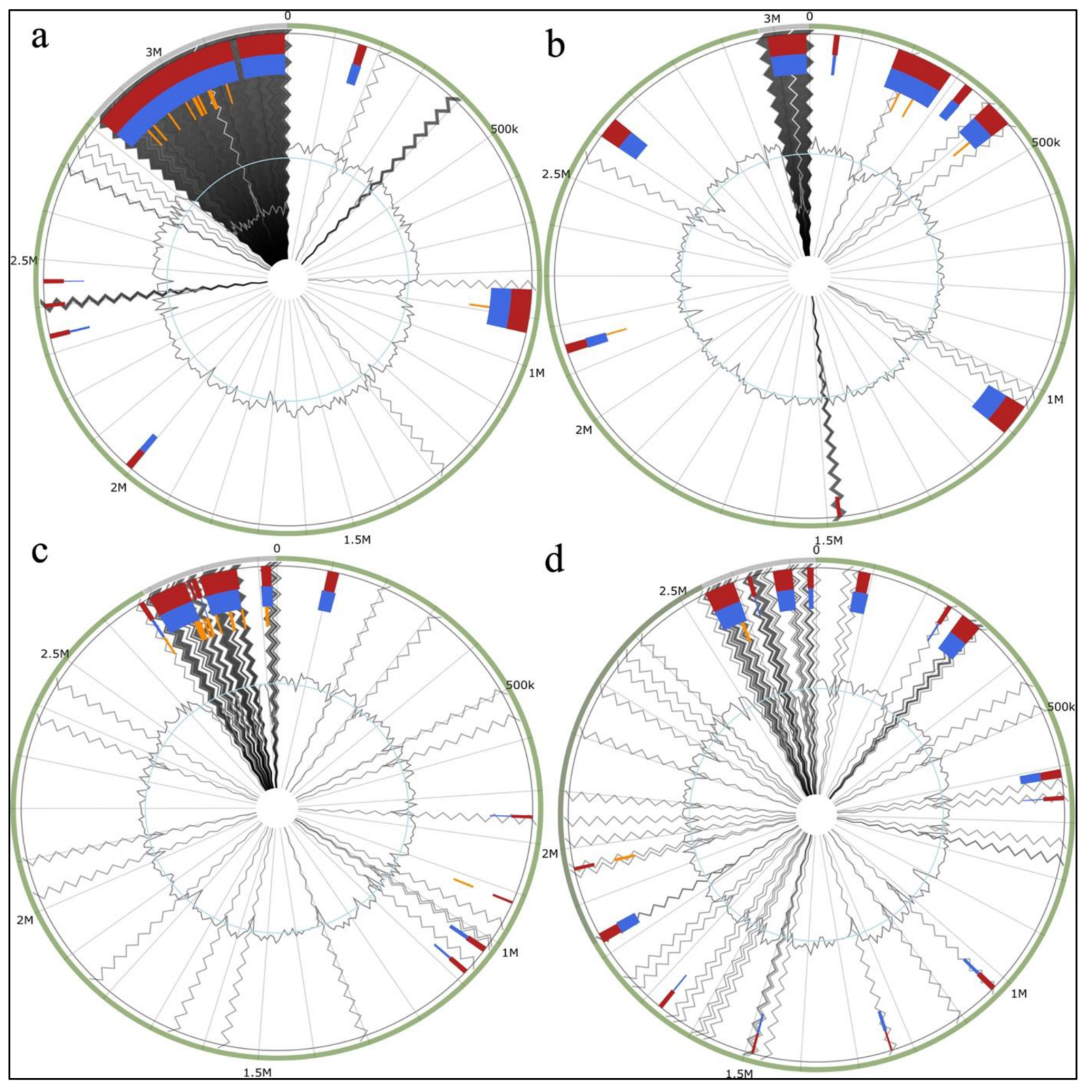
Prediction of genomic islands (GIs) in the *E. faecalis* and *E. lactis* strains. (**a**) L4_6M (**b**) LTM3 (**c**) L16_61 (**d**) LTM3

Here, the GIs of the isolates were identified using the online webserver IslandViewer 4 (Bertelli et al. 2017). The location of GIs in each genome was visualized, which revealed that the number of GI genes in each genome is 291, 346, 428, and 548 for LTM1, L16_61, LTM3, and L4_6M, respectively, indicating that the *E. faecalis* isolates have more GI genes in their genomes than the *E. lactis* isolates (Fig. 3). Very few genes were annotated as virulence and resistance factors, with no gene annotated as virulence/resistance factor in LTM3. At the same time, LTM1 contains only one gene resistance to organic hydroperoxide. L16_61, conversely, contains organic hydroperoxide resistance protein, arsenic resistance proteins (*ACR3*), and cadmium resistance proteins. L4_6M contains GI genes related to cobalt-zinc-cadmium resistance (*CzcD*), and two tetracycline resistance proteins (*Tet*(M) and *TetB*(P). In addition, most of the genes were annotated as hypothetical proteins (Tables S7-S10). This result indicates that *E. faecalis* have more GI genes than *E. lactis*; hence, there is a tendency for increased HGT in these species and a continuous increase in their genetic diversity. In support of our claim, Raven et al. (2016) discussed the contribution of mobile genetic elements, such as plasmids and prophages, to the diversity of *E. faecalis*. The study compares 18 *E. faecalis* strains and demonstrates these elements’ role in shaping the species’ gene content. This supports the idea that *E. faecalis* has more GI genes, which can facilitate HGT and increase genetic diversity. Another study by Anderson et al. (2016), highlights the proficiency of *E. faecalis* in the exchange and transfer of virulence and resistance genes through HGT. The study emphasizes the importance of horizontal gene transfer in the evolution and adaptation of *E. faecalis*. This further supports the claim that *E. faecalis* tends to increase HGT and genetic diversity.

The development of genome sequencing has promoted the realization that HGT is a major evolutionary force reshaping bacterial genomes and, therefore, influencing bacterial adaptation. Except for LTM3, which has no gene annotated in virulence or resistance, HGT does not determine key pathogenicity determinants in this isolate. However, in the other isolates harboring genes related to virulence and resistance factors, further studies should be conducted to identify why HGT can transfer the prevailing genes in these isolates, and their functions in these isolates should be explicitly identified.

Using the perfect and strict hits only, CARD analysis revealed 13 hits with one perfect and 12 strict hits (Table 2). *E. faecalis* L4_6M and LTM3 carried genes including *dfrE*, *vanW*, *vanT*, and the efflux gene *efrA*, which is typically located on the plasmid. The *dfrE* gene is located on a transposon, allowing it to mobilize within the bacterial genome or potentially transfer to other bacteria, while the *vanW* and *vanT* genes are often carried on plasmids or integrated into the chromosome. In addition, L4_6M also encodes the *tet*(M) gene, which is carried on the transposons and confers resistance to tetracycline. The presence of the *dfrE* gene in *E. faecalis* genomes suggests that these bacteria may exhibit resistance to trimethoprim, making it more challenging to treat infections caused by these strains (Sirichoat et al. 2020). Vancomycin-resistant enterococci are a major concern in healthcare settings. The *vanW* and *vanT* genes are part of larger gene operons, including *vanB* and *vanG* operons. It is of paramount importance to know that vancomycin-resistant isolates must show the presence of the 6 genes in the van cluster (*vanX*,* vanH*,* vanW*,* vanY*,* vanS*, and *vanR*) before they can elicit resistance, but none of the isolates in this study meet this criterion to be regarded as vancomycin-resistant isolates. These gene operons encode enzymes that modify peptidoglycan precursors, reducing their affinity for glycopeptide antibiotics like vancomycin (Patiño et al. 2002). Vancomycin is a last-resort antibiotic used to treat severe infections caused by Gram-positive bacteria. The *efrA* gene is an efflux pump gene that confers resistance to various antibiotics, including fluoroquinolones and chlorhexidine. This gene allows bacteria to pump out antibiotics, reducing their effectiveness. Therefore, the presence of this gene suggests that these strains may exhibit multidrug resistance, making them more difficult to treat with commonly used antibiotics (Kumar et al. 2019). The *tet*(M) gene is associated with resistance to tetracycline, a broad-spectrum antibiotic commonly used to treat many bacterial infections. This gene’s presence limits the antibiotic’s effectiveness in treating infections caused by these bacteria (Yang et al. 2019). These antibiotic-resistant genes in *E. faecalis* genomes pose a significant challenge for human health. These genes confer resistance to important antibiotics, limiting treatment options for infections caused by these bacteria. The spread of these resistant genes among *E. faecalis* strains and their potential transfer to other bacterial species further exacerbates the problem of antibiotic resistance. Implementing effective infection control measures and developing alternative treatment strategies to combat the spread of antibiotic resistance (Al-turfi and Hussein 2022; Song et al. 2021).

On the other hand, both *E. lactis* strains carried genes conferring antibiotics, which differ from those of the *E. faecalis* strains. The *E. Lactis* strains genomes encoded the *vanY*, which is one of the gene clusters in the vancomycin-resistant operon, and the *AAC(6”)-Ii* genes, which is an aminoglycoside antibiotics. The *vanY* gene is found on mobile genetic elements such as plasmids, facilitating its transfer between bacteria and the AAC(6”)-Ii gene is typically located on transposons. Furthermore, the *E. lactis* genome encoded the *msrC* gene, the only perfect hit by the CARD analysis (Table 2) and usually found on mobile elements, encoding for resistance to erythromycin antibiotics. Commensal bacteria, including *E. lactis*, can acquire antibiotic resistance genes, such as AAC(6”)-Ii and *msrC*, which enable them to survive and maintain microbial homeostasis in the lower intestinal tract (Szmolka and Nagy 2013). This acquisition of resistance genes by commensal bacteria raises concerns as they can serve as reservoirs of resistance genes that can potentially be transferred to pathogenic bacteria. In addition, the presence of resistance genes in *E. lactis* genomes suggests the potential for horizontal gene transfer between different bacterial species, including pathogens. This transfer of resistance genes can contribute to the spread of antibiotic resistance in the environment and pose a risk to human health. The presence of the *vanY* gene alone in the *E. lactis* genomes cannot confer resistance to vancomycin. Hence, these isolates cannot be regarded as being vancomycin resistant. The presence of this gene in the *E. lactis* genomes suggests the possibility of horizontal gene transfer between enterococcal species, including pathogenic strains. In their comparative genomics studies, Lu et al. (2023) also identified the presence of resistance genes, including AAC(6”)-Ii and *msrC*, in *E. lactis* genomes. This is similar to what we observed in this study. They further suggest that *E. lactis* may be an alternative to *E. faecalis* for use in the food industry due to their lower number of ARGs. Similarly, Choi et al. (2024) also reported the presence of antibiotic-resistance genes in their comparative pangenome analysis study on *E. faecium* and *E. lactis*. The findings revealed fewer resistance genes in the *E. lactis* genomes compared to the *E. faecium* genomes, which are similar to our findings, which show fewer resistance genes in *E. lactis* genomes when compared to the *E. faecalis* genomes.

**Table 2:** CARD antibiotic resistance gene analysis

	RGI Criteria	ARO Term	AMR Gene Family	Drug Class	Resistance Mechanism	% Identity
L4_6M	Strict	dfrE	trimethoprim resistant dihydrofolate reductase dfr	diaminopyrimidine antibiotic	antibiotic target replacement	98.8
	Strict	vanW gene in vanG cluster	vanW, glycopeptide resistance gene cluster	glycopeptide antibiotic	antibiotic target alteration	33.2
	Strict	vanT gene in vanG cluster	glycopeptide resistance gene cluster, vanT	glycopeptide antibiotic	antibiotic target alteration	34.8
	Strict	efrA	ATP-binding cassette (ABC) antibiotic efflux pump	macrolide antibiotic, fluoroquinolone antibiotic, rifamycin antibiotic	antibiotic efflux	99.7
	Strict	tet(M)	tetracycline-resistant ribosomal protection protein	tetracycline antibiotic	antibiotic target protection	94.7
L16_61	Perfect	msrC	msr-type ABC-F protein	macrolide antibiotic, streptogramin antibiotic, streptogramin B antibiotic	antibiotic target protection	100
	Strict	AAC(6’)-Ii	AAC(6’)	aminoglycoside antibiotic	antibiotic inactivation	99.5
	Strict	vanY gene in vanB cluster	vanY, glycopeptide resistance gene cluster	glycopeptide antibiotic	antibiotic target alteration	34.5
LTM1	Strict	AAC(6’)-Ii	AAC(6’)	aminoglycoside antibiotic	antibiotic inactivation	98.9
	Strict	vanY gene in vanB cluster	vanY, glycopeptide resistance gene cluster	glycopeptide antibiotic	antibiotic target alteration	34.5
LTM3	Strict	dfrE	trimethoprim resistant dihydrofolate reductase dfr	diaminopyrimidine antibiotic	antibiotic target replacement	98.2
	Strict	vanT gene in vanG cluster	glycopeptide resistance gene cluster, vanT	glycopeptide antibiotic	antibiotic target alteration	34.8
	Strict	efrA	ATP-binding cassette (ABC) antibiotic efflux pump	macrolide antibiotic, fluoroquinolone antibiotic, rifamycin antibiotic	antibiotic efflux	99.3

### Pangenome and comparative genomics analysis

### Pangenome analysis of South African *E. faecalis* species

Forty-nine South African *E. faecalis* genomes and the reference genome were downloaded from the NCBI database. The assemblies, location, and isolation source are shown in Table S11. The sizes of core and dispensable genomes were estimated using pangenome analysis via two distinct pipelines, Roary and Anvi’o. Roary analysis suggested there were 2157 core genes (27.1%), 1164 shell genes (14.6%), and 4638 cloud genes (58.3%) out of 7959 genes in 52 South African *E. faecalis* genomes (Table 3). Core genomes of similar size were reported in the pangenome analysis of 111(*n* = 2007) *E. faecalis* genomes by Hochstedler-Kramer et al. (2023) and 2026 (*n* = 2068) *E. faecalis* genomes by Pöntinen et al. (2021). The genome group is open, indicating that additional data input will alter the proportion of the core genome and that new orthogroups will be discovered.

**Table 3:** Roary summary statistics of the 52 E. faecalis pangenome analysis

Core genes	(99% <= strains < = 100%)	1784
Soft core genes	(95% <= strains < 99%)	373
Shell genes	(15% <= strains < 95%)	1164
Cloud genes	(0% <= strains < 15%)	4638
Total genes	(0% <= strains < = 100%)	7959

**Fig. 4:**
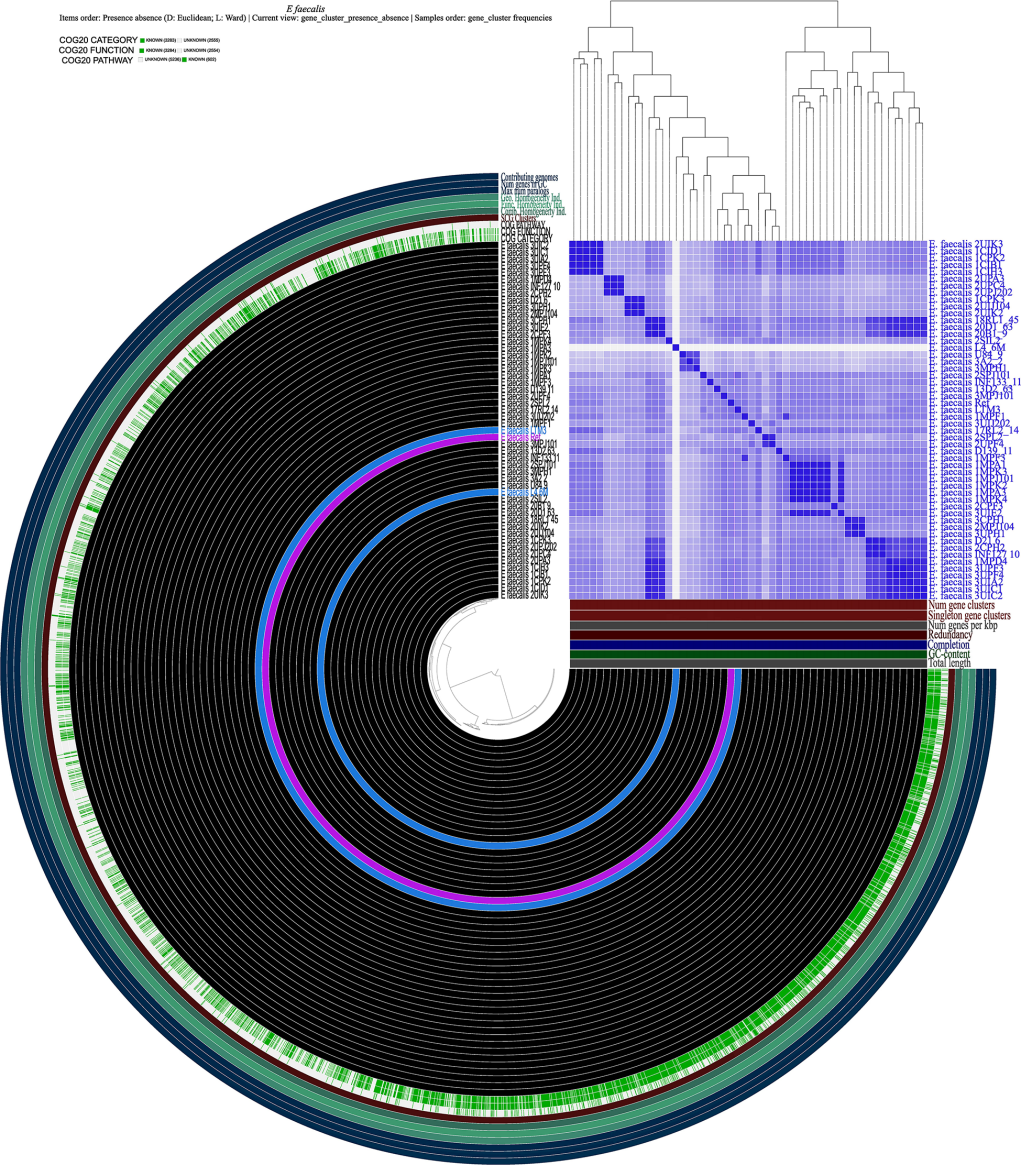
Pangenome analysis. Clustering of genomes based on the presence/absence patterns of 5,832 pangenomic clusters in 51 *E. faecalis* genomes from South Africa and *E. faecalis* reference genome. The genomes are organized in radial layers as core, unique, and accessory gene clusters [Euclidean distance; Ward linkage] defined by the gene tree in the center. The layers represent individual genomes organized by their phylogenomic relationships based on core genes. The blue layers represent the genomes obtained in this study, while the red layer is the reference genome. In the layers, dark colors indicate the presence of a gene cluster, and light color indicates its absence. Average Nucleotide identity values among different genomes were represented on a heatmap determined from the high similarity (purple) and low similarity (white). The figure was constructed using Anvi’o pangenome workflow (http://merenlab.org/software/anvio/)

A circular graph was created via Anvi’o containing information on gene numbers in gene clusters, the maximum number of paralogs, genomic homogeneity index, functional homogeneity index, combined homogeneity index, and single copy gene (SCG) clusters (Fig. 4). Despite both Roary and Anvi’o approaches using the MCL algorithm to identify clusters, the Anvi’o pangenome workflow identified fewer gene clusters (5838), which may have led to fewer core genes. The differences might be due to different ways to establish orthologs of protein clusters. Roary divides groups of homologous sequences into paralogs and orthologs using conserved gene neighborhood information, while Anvi’o clusters orthologs based on the homology and synteny of genes (Zhou et al. 2021). The core genome observed from the Roary and Anvi’o analysis suggests that the *E. faecalis* species may contain more accessory genes critical for adaptation to different environments and survival (Tables S12 and S13). Similar findings were reported by Bakshi et al. (2016), who conducted a pan-genomic analysis of uncharacterized *E. faecalis* strains and identified the core genome. They found that the core genome of *E. faecalis* strains represents 68.7% of the average number of genes per genome. However, when considering it as a fraction of the pan-genome size, it is relatively small at 29.04%. This suggests that *E. faecalis* may contain more accessory genes critical for adaptation to different environments and survival. This finding is further supported by the study conducted by He et al. (2018), where they constructed the core- and pan-genomes of *E. faecalis* based on the families of homologous genes. They found that the core genome of *E. faecalis* is relatively small compared to the pan-genome. Furthermore, an earlier study by Paulsen et al. (2003) revealed that more than a quarter of the genome of *E. faecalis* V583, a vancomycin-resistant clinical isolate, consists of probable mobile or foreign DNA. This suggests that a significant portion of the genome is not part of the core genes.

### Comparative analysis of ARGs in South African *E. faecalis* genomes

In total, 267 ARG hits and variants were detected in the collected genomes based on CARD analysis using only the perfect and strict hits, which resulted in 20 perfect hits and 247 strict hits. The frequency of ARGs was found to be in the range of 3–14 genes (Table S14). A maximum of 14 ARGs were found in the genome of *E. faecalis* 2SIL2, 12 ARGs in 3UIJ202, 11 ARGs in 3UIC2 and 3UIC1, and 8 ARGs in D21_6. Two isolates had 7, 11 isolates had 6, eleven had 5, nine had 4, and twelve had 3 ARGs (Table S14).

Moreover, glycopeptide, diaminopyrimidine, tetracycline, and multi-drug mediated resistance genes were present in most of the isolates (Table S14). All the strains were carrying the diaminopyrimidine and glycopeptide antibiotics resistant class specifically, the trimethoprim-resistant dihydrofolate reductase (*dfrE*) gene for the diaminopyrimidine class, and *vanT*,* W*,*Y* for the glycopeptide class. All isolates were carrying efflux-associated ARGs (*efrA*). Tetracycline-resistant genes tet(M) and tet(45) were found in 38 and 5 strains respectively. Based on this result, we can conclude that the most abundant antibiotic-resistant genes in the South African *E. faecalis* strains are those resistant to trimethoprim (*dfrE*), tetracycline (*tet(M)*), glycopeptide antibiotics (*vanT*), and the multi-drug resistant (*efrA*) gene. The *efrA* gene is commonly found in Gram-negative bacteria and is associated with resistance to efflux pump inhibitors (Hu et al. 2013). The *tetM* gene, which confers resistance to tetracycline, is frequently detected in *Staphylococcus aureus* and *Streptococcus* species (Ding et al. 2016; Hui-Ling Ong et al. 2017). The *dfrE* gene is associated with resistance to trimethoprim and is reportedly detected in various bacterial species (Lienen et al. 2022).

### Virulence-associated genes comparative analysis of the *E. faecalis* genomes

**Fig. 5:**
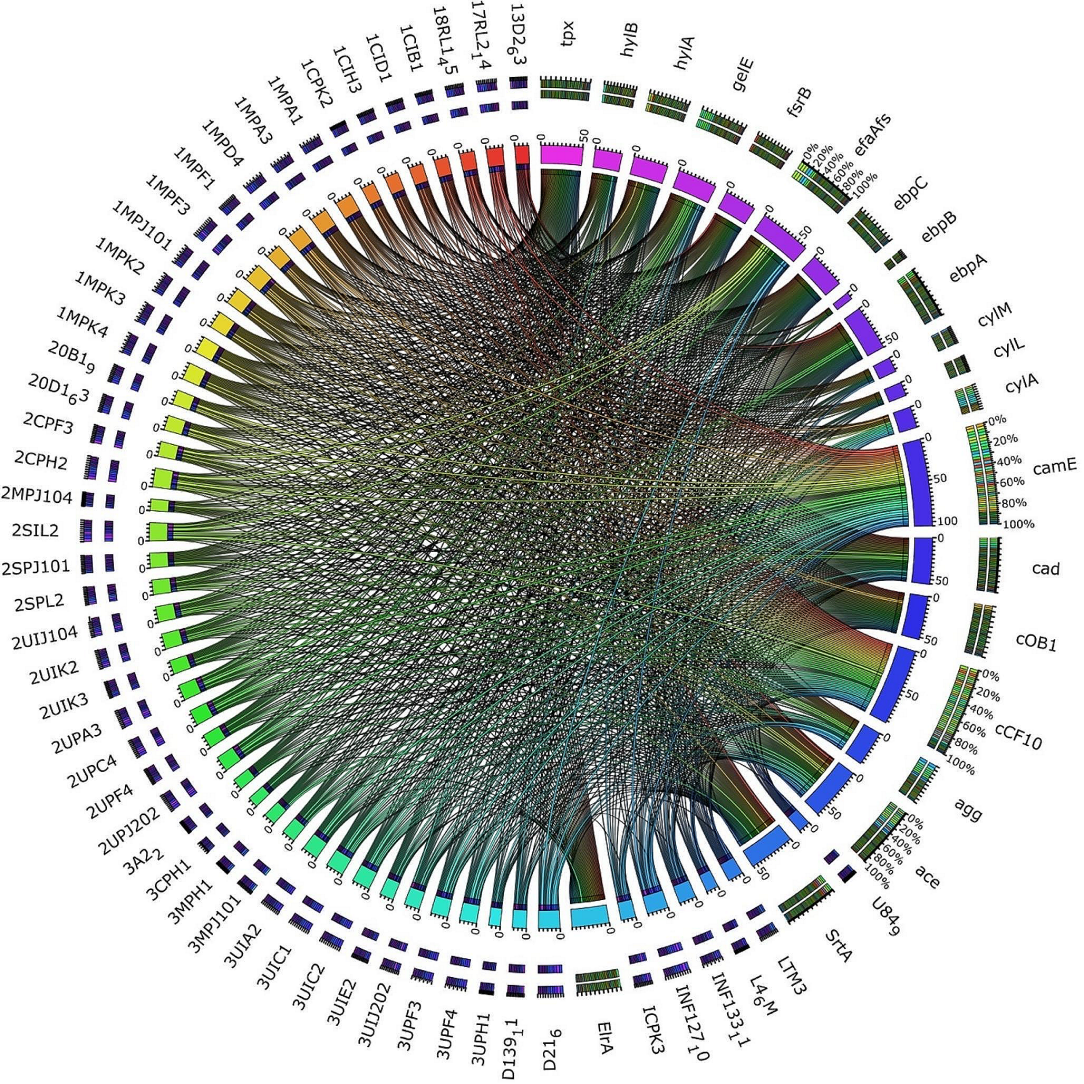
Circos plot of the virulence genes in all E. faecalis genomes of South African origin from the NCBI database

*E. faecalis* virulence gene contents greatly influence the degree of pathogenicity of these microbes. In this study, 961 virulence genes were identified in all the South African *E. faecalis* strains (Fig. 5), with the smallest; *ebpB* gene occurring 9 times and the highest occurrence being the *camE* gene, totaling 105 in all the strains (Fig. 5). In general, the genes identified in this study encompass a variety of functions, including those linked to biofilm formation (*SrtA*), cell-cell communication (*cCF10*, *cOB1*, and *cad*), pilus biogenesis, and adhesion (*ebpA* and *efaAfs*), as well as combating oxidative stress (*tpx*). These genes were identified across all strains examined. Two extracellular hyaluronidase genes *hylA* and *hylB* that evade the phagocytosis process with macrophage persistence of host, were identified with *hylA* identified in 40 strains while *hylB* was identified in 32 strains. Some strains also identified other genes, including the *agg* gene associated with biofilm formation, *ebpBC* genes associated with pilus biogenesis and biofilm formation, and the ace gene associated with collagen adhesion. Other virulence factors included genes associated with macrophage persistence (*elrA*), hydrolysis of gelatin (*gelE*), the quorum sensing linked *fsrB* gene, and the cytolysin toxin-producing genes *cylA*, *cylL*, and *cylM*.

In *E. faecalis*, several virulence factors play pivotal roles in establishing infections. One such factor is the surface protein antigen A (*SrtA*), which is involved in anchoring surface proteins to the cell wall, facilitating the adherence of the bacterium to host tissues and biofilm formation. The ability to adhere to host tissues is crucial for initiating infection. Additionally, the endocarditis and biofilm-associated pili (ebp) are significant virulence factors, with *ebpA* being a subunit of ebp pili. These pili aid in adherence to host tissues and biofilm formation, which enhances the bacterium’s persistence and antibiotic resistance. The enterococcal surface protein antigen (*EfaAfs*) also contributes to adherence and biofilm formation, enabling *E. faecalis* to evade host defenses. Thioredoxin peroxidase (*tpx*) is an antioxidant enzyme that protects the bacterium from host-generated oxidative stress, further supporting its pathogenicity. Hemolysin genes *hylA* and *hylB* cause red blood cell lysis and tissue damage, bolstering *E. faecalis’* pathogenicity. Lastly, the aggregation substance (*agg*) and *ebpBC* operon contribute to adherence, biofilm formation, and overall virulence, emphasizing their importance in the pathogenesis of *E. faecalis* infections. These factors collectively underscore the multifaceted nature of *E. faecalis* virulence mechanisms and have been reported in various studies (Barbosa et al. 2010; Eaton and Gasson 2001; Haghi et al. 2019; Zheng et al. 2017). Similarly, these genes were found in the genome sequences of the *E. faecalis* strains in the current study.

To elaborate on the discussion, other resistance and virulence genes common to the enterococcus are not identified in this study but are pertinent to the epidemiology associated with these organisms. Enterococci are known for their diverse virulence factors contributing to their pathogenicity. Hospital-acquired *E. faecium* strains have been reported to possess high-level resistance to antibiotics like ampicillin and ciprofloxacin (Choi et al. 2024), along with an enrichment of putative virulence genes such as *esp*, which is involved in biofilm formation and genes encoding pilus-like structures and cell wall-anchored *LPxTG* surface proteins (Hendrickx Antoni et al. 2009). Furthermore, the *epa* gene cluster in *E. faecalis* is crucial for polysaccharide biosynthesis, shape determination, biofilm formation, and virulence in mouse models of peritonitis (Teng et al. 2009). Studies have also highlighted the prevalence of virulence determinants and antibiotic-resistance genes in enterococci isolated from hospitalized patients, emphasizing the importance of understanding the genetic makeup of clinical isolates (Haghi et al. 2019). The *Fsr* quorum-sensing system in *E. faecalis* has been shown to modulate the surface display of the collagen-binding MSCRAMM Ace through the regulation of gelatinases, further underlining the intricate regulatory mechanisms governing virulence in these bacteria (Pinkston Kenneth et al. 2011). Moreover, the incidence of virulence determinants in clinical *E. faecalis* and *E. faecium* isolates has been documented, with a particular focus on genes encoding virulence factors involved in biofilm formation, such as enterococcal surface protein, aggregation substance, and gelatinase (Strateva et al. 2016). Enterococci isolated from urinary tract infections have been studied for their virulence factors and antimicrobial resistance patterns, shedding light on the significance of these bacteria as nosocomial pathogens (Suchi et al. 2017). The *E. faecalis* MSCRAMM Ace has been identified as a collagen-binding adhesin, crucial for host cell adherence, and implicated in conditions like endocarditis (Liu et al. 2007). Additionally, *E. faecium* strains exhibit strain-specific collagen binding mediated by *Acm*, a member of the MSCRAMM family, emphasizing the diversity in adhesins among enterococci (Nallapareddy et al. 2003). Bacteriocin production by enterococci has been shown to augment niche competition in the gastrointestinal tract, highlighting the role of bacteriocins in microbial interactions within the host (Kommineni et al. 2015). The presence of a family of putative MSCRAMMs in *E. faecalis* underscores the importance of surface proteins in mediating host-pathogen interactions and colonization (Sillanpää et al. 2004). Comparative genomic analysis has revealed a significant enrichment of mobile genetic elements and genes encoding surface structure proteins in hospital-associated clonal complex 2 *E. faecalis* strains, suggesting a link between genetic elements and virulence potential (Solheim et al. 2011). The role of fibrinogen-binding MSCRAMMs in *E. faecalis* has been elucidated, emphasizing the importance of these adhesins in host tissue adherence and infection establishment (Sillanpää et al. 2009). Furthermore, the collagen-binding MSCRAMM Ace has been characterized structurally and functionally, highlighting its significance in the pathogenesis of *E. faecalis* infections (Rich et al. 1999). Enterococci and other gram-positive pathogens utilize surface proteins like MSCRAMMs to adhere to host tissues, facilitating infection initiation (García-Solache and Rice Louis 2019). The adaptability of enterococcus to its environment is underscored by the presence of MSCRAMMs that aid in host tissue adhesion and colonization. The *LPxTG*-type surface proteins found in enterococci, including pili and MSCRAMMs, play a crucial role in mediating bacterial attachment to host tissues (Geraldes et al. 2022). The diverse array of surface proteins, including adhesins and pili, found in enterococci enables these bacteria to colonize and cause infections in various host environments (Sillanpää et al. 2009).

## Conclusion

This study provided insights into the distribution of ARGs and virulence factors in the genomes of isolated *E. lactis* and South African *E. faecalis*. Despite the limited sample size of four new genomes, our analysis revealed a diverse array of ARGs in the South African *E. faecalis* genomes, including those conferring resistance to trimethoprim (*dfrE*), tetracycline (*tet*(M)), glycopeptide antibiotics (*vanT*), and the multi-drug resistance gene (*efr*A). The presence of the *efrA* gene, typically associated with Gram-negative bacteria, suggests potential horizontal gene transfer events. Virulence-associated genes such as *SrtA*, ebp pili (*EbpA*), *EfaAfs*, *tpx*, *hylA*, and *hylB* were also identified, highlighting the pathogenic potential of *E. faecalis*. These findings underscore the importance of continued genomic surveillance and research to understand the mechanisms of antibiotic resistance and virulence in Enterococcus species. Although our conclusions are based on a limited number of new genome sequences, they contribute to a broader understanding of ARGs and virulence factors in Enterococcus species. The implications extend to clinical decision-making, infection control practices, and the development of therapeutic interventions. Furthermore, fewer ARGs and virulence genes in *E. lactis* support its potential as a safer alternative to *E. faecalis* in the food industry.

Future studies should focus on the functional validation of the roles of identified genes and expand genomic analyses to include a larger and more diverse collection of Enterococcus isolates from different regions. This approach will strengthen our understanding and inform strategies to combat antibiotic resistance, advocating for a one health approach that recognizes the interconnected nature of human, animal, and environmental health.

## Electronic supplementary material

Below is the link to the electronic supplementary material.

Supplementary Material 1

Supplementary Material 2

## Data Availability

Raw genome sequencing reads are deposited in NCBI under Genbank accession numbers JAWJDK000000000, JAWKDV010000000, JAWJDI000000000, and JAWJDJ000000000 for L4_6M, L16_61, LTM1, and LTM3 respectively and BioProject numbers PRJNA1027583, PRJNA1027576, PRJNA1027578, and PRJNA1027580, respectively.
