# Supplementary figures and images for: Genomic diversity, antibiotic resistance, and virulence in South African Enterococcus faecalis and Enterococcus lactis isolates
Source: World J Microbiol Biotechnol. 2024 Aug 5;40(10):289. doi: 10.1007/s11274-024-04098-5 (PMC11300488; doi:10.1007/s11274-024-04098-5)

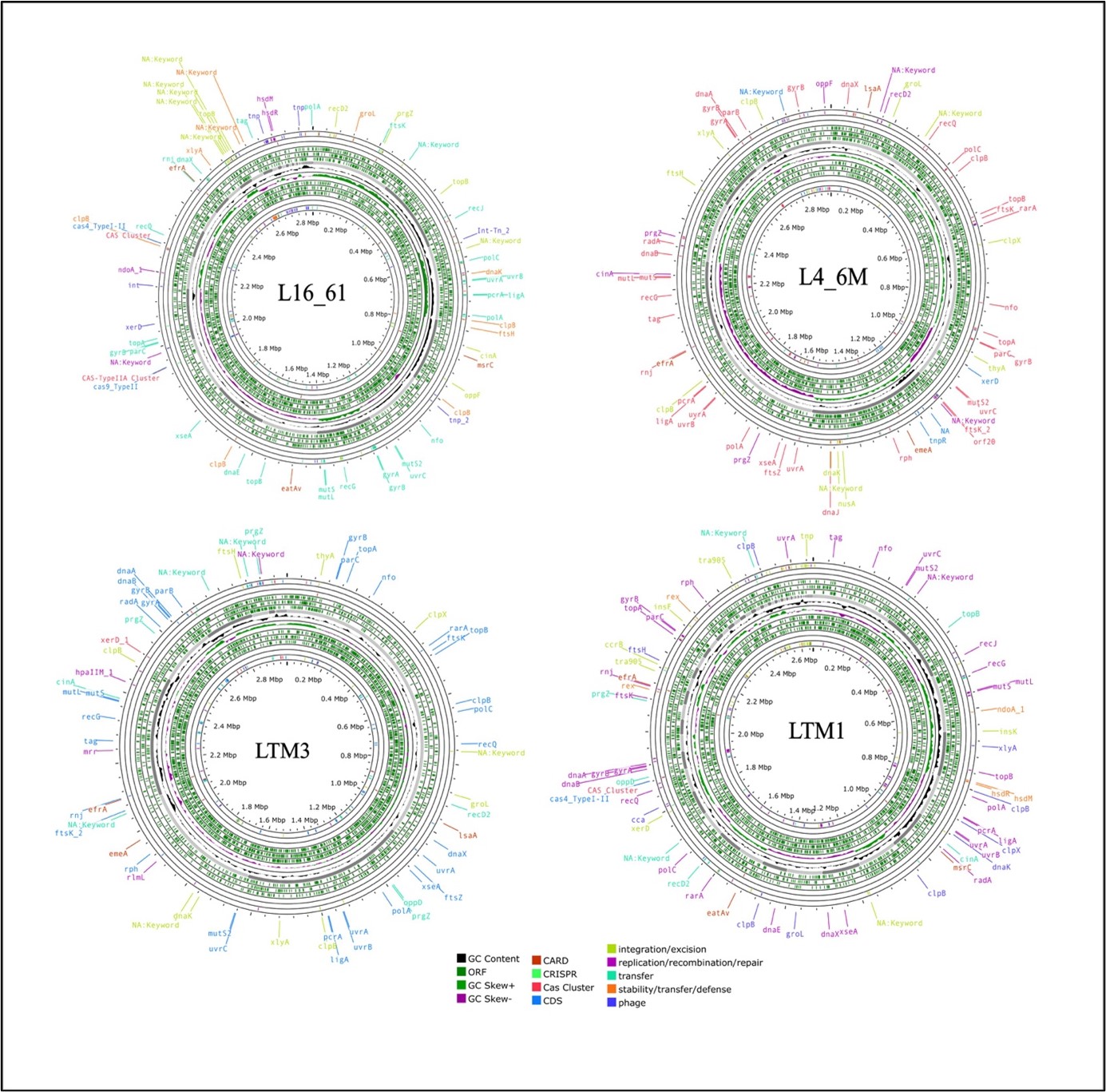

Supplement: Supplementary file 2 — Supplementary Material 2 [file 11274_2024_4098_MOESM2_ESM.jpg]
